# Sintilimab-induced diabetes mellitus and thyroid dysfunction in patient with gastric adenocarcinoma: A case report and literature review

**DOI:** 10.1097/MD.0000000000042490

**Published:** 2025-05-16

**Authors:** Ying Zan, Yedong Wei, Wenxue Zhang, Xiaolu Gao, Jigang Si

**Affiliations:** a Department of Pharmacy, Zibo Central Hospital, Zibo, China; b Department of Pharmacy, Zhangdian Traditional Chinese Medicine Hospital, Zibo, China; c Department of pharmacy, Jinshan Town Health Center, Zibo, China.

**Keywords:** DIRE, ICI-induced diabetes mellitus, ICI-induced thyroid dysfunction, irAEs, sintilimab

## Abstract

**Rationale:**

Immune checkpoint inhibitors bring hope to cancer patients but may also lead to severe immune-related adverse events (irAEs). Although irAEs during treatment are well-characterized, delayed immune-related events (DIRE) remain underreported. Here, we report a case of sintilimab-induced delayed immune-related diabetes mellitus, accompanied by ICI-related thyroid disease (ICI-TD). Cases involving both ICI-TD and ICI-related diabetes mellitus (ICI-DM) are also relatively rare. This study systematically aggregates dual endocrine irAEs to provide valuable insights for clinical practice.

**Patient concerns:**

A 60-year-old Chinese male diagnosed with gastric adenocarcinoma received a multimodal treatment regimen consisting of sintilimab, chemotherapy, and apatinib. He completed 3 cycles of chemotherapy and 4 cycles of sintilimab. Due to disease progression, sintilimab was discontinued, but apatinib was continued for an additional 1 month. No further antitumor therapy was administered afterward. Four months later, he was admitted to the emergency department due to persistent nausea, vomiting, and abdominal pain.

**Diagnoses:**

Thyroid dysfunction induced by sintilimab was identified during treatment. His laboratory tests contributed to the diagnosis of diabetes ketoacidosis. Fulminant type 1 diabetes mellitus attributed to sintilimab met diagnostic criteria: plasma glucose 42.01 mmol/L, glycated hemoglobin 7.5%, C-peptide <0.02 µg/L, and negative islet autoantibodies.

**Interventions:**

Levothyroxine replacement therapy was initiated for hypothyroidism, whereas diabetes ketoacidosis during hospitalization required intensive insulin therapy combined with fluid resuscitation.

**Outcomes:**

The patient exhibited persistent blood glucose fluctuations during hospitalization, including 2 hypoglycemic episodes. Post-treatment stabilization required basal-bolus insulin at discharge, with continued levothyroxine for hypothyroidism.

**Lessons:**

We report a rare case of concurrent ICI-TD and ICI-DM following sintilimab therapy. This case underscores the potential for DIRE, with onset occurring months posttreatment. Combined with a systematic review of existing cases, this study provides critical insights into surveillance strategies and pathogenesis of irAEs.

## 1. Introduction

In recent years, immune checkpoint inhibitors (ICIs) have emerged as powerful new drugs for the treatment of cancer. Immune checkpoints play a crucial role in maintaining immune tolerance and preventing autoimmune diseases. However, their inhibition by ICIs paradoxically enhances antitumor immunity while predisposing to autoimmune toxicities, reflecting the dual-edged role of ICIs in cancer therapy.

Multiglandular immune-related adverse events (irAEs) are generally associated with combined ICIs, whereas those induced by single ICI are uncommon.^[1]^ However, with the widespread use of ICIs, reports of multiglandular irAEs associated with individual drugs have become more frequent.

Although the prevalence of ICI-induced diabetes mellitus (ICI-DM) is only 0.9%,^[2]^ it can progress rapidly and become life-threatening, unlike other endocrine disorders. Up to 44% of patients experience other endocrine issues before or concurrently with diabetes, with the thyroid being the most commonly affected.^[2,3]^ This often leads to permanent hypothyroidism, which requires lifelong thyroxine supplementation, and in severe cases, can result in a thyroid crisis. However, thyroid issues often have a considerable latency period, are nonspecific in the early stages, and are difficult to identify. This significantly impacts the quality of life for patients and can disrupt ongoing antitumor therapy and prognosis.

The prescribing information often mention that delayed immune-related events (DIRE) may occur after discontinuation of ICIs, but in practice, reports of such cases are rare. DIRE was defined as new irAE manifesting ≥ 90 days after discontinuation of immunotherapy.^[4]^ For patients with prior ICIs exposure, heightened vigilance for DIRE is critical to prevent misattribution-related harms: delayed autoimmune disease management and inappropriate discontinuation of beneficial therapies. In this context, we report a case of fulminant type 1 diabetes (FT1DM) and thyroid dysfunction with gastric adenocarcinoma induced by sintilimab. To our knowledge, this is a rare case of sintilimab-induced ICI-DM and ICI-induced thyroid dysfunction (ICI-TD). Additionally, the uniqueness of the case lies in the fact that his diabetes developed 4 months after discontinuing sintilimab.

We also review previous reports of these concurrent irAEs, aiming to assist clinicians in the early detection, diagnosis, prevention, and management of these conditions to reduce their severity and improve patient outcomes.

## 2. Case presentation

### 2.1. Initial diagnosis of gastric cancer

We presented a 60-year-old Chinese male patient with no prior history of diabetes mellitus or thyroid dysfunction, and a medical history of hypertension managed with regular nifedipine and valsartan. In February 2023, gastroscopy revealed a gastric antral lesion confirmed as poorly differentiated adenocarcinoma. Intraoperative findings showed extensive peritoneal metastases (pT4NxM1), prompting palliative gastrojejunostomy due to unresectability.

### 2.2. Treatment course and thyroid function changes

**Figure 1.**
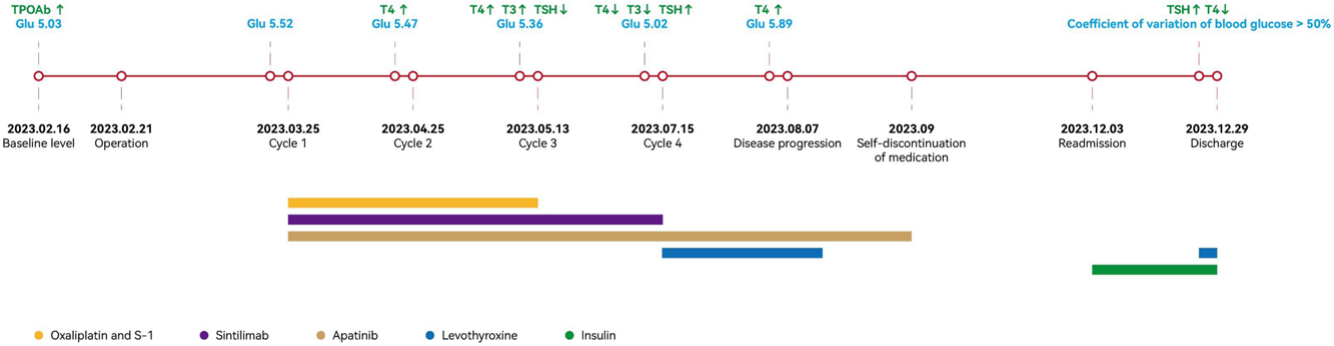
Timeline of patient treatment process.

One month postoperatively, the patient was initiated on the SOX chemotherapy regimen (oxaliplatin + S-1) combined with sintilimab and apatinib (Fig. 1). Prior to initial treatment, the patient’s blood glucose levels and thyroid function were within normal limits, except for elevated thyroid peroxidase antibodies (TPOAb) level. Color Doppler ultrasonography demonstrated multiple discrete nodules without diffuse parenchymal changes.

The patient tolerated the first treatment cycle well. However, laboratory evaluations prior to cycle 2 revealed a marginally elevated T4 level (Table 1). During pre-cycle 3 screening, the patient was found to have developed thyrotoxicosis. Nevertheless, no significant symptoms were observed, requiring no therapeutic intervention, and anticancer therapy proceeded as scheduled.

**Table 1:** Thyroid function test results during hospitalization.

	41 d before the first cycle	20 d after the first cycle	27 d after the second cycle	61 d after the third cycle	23 d after the fourth cycle	149 d after the fourth cycle
TSH	1.38	0.883	0.017↓	34.072↑	2.984	17.592↑
FT3	5.16	N/A	N/A	N/A	N/A	N/A
FT4	14.66	N/A	N/A	N/A	N/A	N/A
T3	N/A	2.23	4.09↑	0.69↓	1.02	1.17
T4	N/A	186.98↑	328.38↑	54↓	278.95↑	59.45↓
TPOAb	23.3↑	N/A	N/A	N/A	N/A	N/A
TgAb	0.2	N/A	N/A	N/A	N/A	N/A
Thyroid ultrasound	multiple nodules，TIRADS-3	N/A	N/A	N/A	N/A	N/A
Levothyrocine	–	–	–	100 μg/d	100 μg/d	100 μg/d

Normal range: FT3 (pmol/L): 3.5 to 6.5; FT4 (pmol/L): 7.01 to 15.96; TPOAb (IU/mL): 0 to 9; TgAb (IU/mL): 0 to 0.4; TSH (mU/L): 0.56 to 5.91; T3 (nmol/L): 1.012 to 2.479; T4 (nmol/L): 69.97 to 152.51.

Abbreviations: FT3 = free triiodothyronine, FT4 = free thyroxine, T3 = triiodothyronine, T4 = thyroxine, TgAb = thyroglobulin antibody, TPOAb = thyroid peroxidase antibody, TSH = thyroid stimulating hormone.

In July 2023, during thyroid function reassessment, laboratory evaluation revealed a T4 level of 54 nmol/L, T3 of 0.69 nmol/L, and TSH of 34.072 mU/L, consistent with overt hypothyroidism. Based on these findings, levothyroxine replacement therapy was initiated at 100 μg daily. During this treatment phase, the patient opted to discontinue the SOX regimen and received sintilimab combined with apatinib as monotherapy.

The August 2023 follow-up evaluation demonstrated persistent elevation of T4 with other thyroid function parameters remaining within normal limits. However, disease progression was identified during the examination, leading to the discontinuation of sintilimab after completing 4 cycles. After that, the patient self-discontinued levothyroxine and continued monotherapy with apatinib for approximately 1 month. Subsequently, the patient underwent no further antitumor therapy.

### 2.3. FT1DM after the discontinuation of sintilimab

Four months after the discontinuation of sintilimab, the patient was readmitted for follow-up treatment due to persistent nausea, vomiting, and abdominal pain. On the second day of admission, the patient presented with altered consciousness and critical blood biochemical values: glucose level was 42.01 mmol/L, urgent blood gas analysis revealed a pH of 6.922, and blood ketone body level was 2+. Given the diagnosis of diabetic ketoacidosis (DKA), immediate symptomatic treatment, including continuous insulin infusion and fluid supplementation, was initiated.

Subsequent tests revealed a glycated hemoglobin level of 7.5%, an insulin level of 0.46 μIU/L at 0 hours, a C-peptide level of <0.02 μg/L at 0 hours. Additionally, the negative islet-related antibodies (Table 2) and the disparity between HbA1c and blood glucose levels indicated an acute disruption of islet function, which was fulfilled Japanese Diabetes Society 2012 criteria for FT1DM.

**Table 2:** Diagnostic laboratory parameters for diabetes and diabetic ketoacidosis.

Laboratory data	Result	Reference range
Sodium	137	136 to 146 mmol/L
Potassium	5.7↑	3.4 to 4.5 mmol/L
PH	6.922↓	7.35 to 7.45
pCO_2_	12.1↓	35 to 45 mm Hg
HCO_3_^-^	2.4↓	22 to 26 mmol/L
Anion gap	29.9↑	8.0 to 16.0 mmol/L
Lactate	3.0↑	0.5 to 1.6 mmol/L
Glucose	42.01↑	3.90 to 6.10 mmol/L
Serum C-peptide	＜0.02↓	1.1 to 4.4 µg/mL
GADA	2.02	0 to 10 IU/mL
IAA	0.48	0.0 to 1 COI
ICA	0.19	0.0 to 1 COI
HbA1c	7.5↑	3.6% to 6.0%
Blood ketone bodies	2+↑	Negative

Abbreviations: GADA = glutamic acid decarboxylase antibody, HbA1c = hemoglobin A1c, IAA = insulin autoantibody, ICA = islet cell antibody.

**Figure 2.**
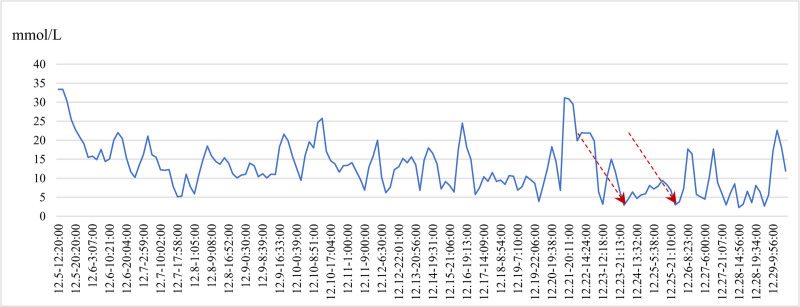
Range of blood glucose fluctuations during hospitalization with 2 hypoglycemic episodes.

The patient’s blood glucose levels were monitored for approximately 1 month during hospitalization. Despite continuous adjustment of the insulin dose based on the patient’s blood glucose levels, the coefficient of variation of glucose remained high at 50%, and the patient experienced 2 severe hypoglycemic episodes (Fig. 2). This was speculated to be related to the nearly complete loss of C-peptide secretion within 1 month after disease onset, indicating the patient had entered a fragile stage of diabetes.

After stabilizing blood glucose levels, the insulin C-peptide release test indicated a complete lack of insulin secretion (Table 3). At discharge, given the patient’s poor islet function, the treatment plan included administering 3 units of rapid-acting insulin before each meal and 6 units of long-acting insulin before bed.

**Table 3:** Islet function before discharge.

Islet function	Result	Reference range
INS0	1.45	2.6 to 24.9 mIU/L
INS1	1.12	
INS2	2.12	
INS3	2.4	
C-P	0.08	1.1 to 4.4µg/L
C1h	0.07	
C2h	0.2	
C3h	0.4	

Abbreviations: C-P = C-peptide; INS = insulin.

Given that all of the patient’s previous blood glucose records were within the normal range, we have reason to suspect that the patient’s current diabetes mellitus is induced by sintilimab.

During this hospitalization, laboratory tests revealed hypothyroidism, prompting the continuation of levothyroxine therapy (100 μg/d) for thyroid hormone replacement. Following this, the patient received Traditional Chinese Medicine (specific therapeutic regimen not documented) and was subsequently lost to follow-up at our institution.

## 3. Methods

Limited data exists on concurrent endocrinopathies in patients receiving ICIs. Therefore, it is essential to consolidate these cases. We conducted a search of English-language case reports on ICI-DM combined with ICI-TD up to May 2024 using Medical Subject Headings (MeSH) terms and keywords from PubMed. Additionally, we performed a secondary search of the bibliographies of all included manuscripts.

A total of 27 reports^[1,5–30]^ involving 30 patients were retrieved. Each case provided the following details: year of publication, patient’s age, gender, tumor type, type of ICI, previous medical history, previous medication history, onset time of diabetes, HbA1c levels, C-peptide levels, presence of DKA, islet autoantibodies, human leukocyte antigen (HLA) genotypes, onset time of thyroid dysfunction, types of thyroid dysfunction, thyroid autoantibodies, other glands involved and medications used (Table 4).

**Table 4:** Detailed case summary of ICI-induced thyroid dysfunction and diabetes (n = 30).

No.	1^[5]^	2^[6]^	3^[1]^	4^[7]^	5^[8]^	6^[9]^	7^[10]^	8^[11]^	9^[12]^	10^[13]^
Publication year	2024	2024	2023	2023	2022	2022	2021	2021	2020	2020
Gender	F	F	M	M	F	F	M	F	M	M
Age (yr)	42	60	53	61	59	59	67	59	85	48
Cancer type	Malignant vaginal melanoma	SCC (urethral)	GE junction adenocarcinoma	SCC (oropharynx)	Lung adenocarcinoma	NSCLC	Melanoma	Melanoma	Cutaneous melanoma	Parotid adenocarcinoma
ICI type	Nivolumab + ipilimumab	Camrelizumab	Pembrolizumab	Teriprizumab	Pembrolizumab	Pembrolizumab	Nivolumab	Ipilimumab+ nivolumab	Pembrolizumab	Nivolumab
Previous medical history	–	–	–	Hypertension and cerebral infarction	Hypertension and acute ischemic stroke	Elevated blood lipids	Chronic sinusitis and thyroiditis	–	–	–
Previous medication history	–	Combined anlotinib	Capecitabine, oxaliplatin	Docetaxel, cisplatin, and tigisay	Aspirin, simvastatin, perindopril, omeprazole，Erlotinib	Statins，carboplatin combined with pemetrexed (5 cycles)	–	–	–	–
Time to diabetes onset (wk)	10	45	6	3	3	18	8	7	7	16
Diabetes antibodies	GADA (−)	GADA (−) IA-2A (−) ICA (−) IAA (−)	GADA (−)	GADA (+) IAA (−)	GADA (+) ICA (−) IAA (−) ZnT8 (−)	GADA (−) IAA (+) ICA (−)	GADA (+)	GADA (−)	GADA (+)	GADA (−) IA-2A (−) ZnT8 (−)
DKA	No	Yes	Yes	Yes	Yes	Yes	No	Yes	No	No
HbA1c (%)	6.7	5.7	7.2	8.3	5.6	8.5	7.1	NR	9	6.4
C-peptide	0.13 ng/mL	<0.3 ng/mL	<0.1 ng/mL	<0.01 nmol/L	＜0.2 ng/mL	<0.02 ng/mL	2.6 ng/mL	<0.1 ng/mL	NR	1.55 ng/mL
HLA	DRB1*04:05- DQB1*04:01	–	–	–	–	–	DRB1*08:02-DQB1*03:02	-	-	DRB1*04:05
Time to thyrotoxicosis onset (wk)	7	–	6, Normal after 100 d of medication	3	–	–	8	7	-	16, Graves’ disease， 100 d later subclinical
Time to hypothyroidism onset (wk)	UK	25	–	–	3	6	12	-	7	-
TD antibodies	TRAb(+) TPOAb(+) TgAb(+)	TPOAb(+) TgAb(+)	TPOAb(−) TRAb(−)	TRAB(−) TPOAb(+) TgAb(+)	TPOAb(+)	TPOAb(+) TgAb(−)	TPOAb(+) TgAb(+)	TPOAb(−) TgAb(−)	TPOAb(−)	TRAb(+) TPOAb(−) TgAb(−)
Therapeutic drug	L-T4 (50 µg/d)	L-T4 (75 µg/d) Normal after 1 mo of medication	Propranolol	–	L-T4 (112 µg/d)	L-T4 (75 µg/d)	–	–	–	Methimazole (2.5 mg/d)
Other glands involved	ICI-induced hypophysitis	Primary adrenal insufficiency (Addison disease)	–	Crohn disease	–	–	Isolated adrenocorti-cotropin	Hepatitis, adrenal insufficien-cy, arthritis and vitiligo	ICI-induced pneumonitis， polymyalgia rheumatica	–
Other Drugs	HC (15 mg/d)	HC	–	Mesalazine	–	–	–	HC	HC	–

No.	11^[14]^	12^[15]^	13^[16]^	14^[17]^	15^[18]^	16^[19]^	17^[20]^	18^[21]^	19^[22]^	20^[23]^
Publication year	2020	2020	2019	2019	2019	2019	2019	2019	2018	2018
Gender	M	M	F	M	M	M	F	F	F	F
Age (yr)	75	78	49	55	52	61	77	43	56	68
Cancer type	SCC (lung)	High-grade neuroendocrine rectal tumor	Lung adenocarcinoma	Carcinoma of bladder	Melanoma	NSCLC	Ulcerated acral melanoma	Melanoma	Lung adenocarcinoma	Renal cell carcinoma
ICI type	Durvalumab	Nivolumab	Durvalumab	Durvalumab	Pembrolizumab	Pembrolizumab	Pembrolizumab	Nivolumab	Nivolumab	Nivolumab
Previous medical history	Chronic obstructive pulmonary disease, hypertension, dyslipidemia, pulmonary embolism， benign prostatic hyperplasia	Prostate cancer, hypertension，gout	–	Arterial hypertension, psoriasis, subclinical hyperthyroidism	–	–	Asthma， paroxysmal atrial fibrillation, gastro-esophageal reflux disease and hypertension	–	–	Subclinical hypothyroidism
Previous medication history	–	–	–	Enalapril	–	–	Inhaled corticosteroids and salbutamol	–	Pemetrexed, cisplatin	Sunitinib， interferon α combined with axitinib for 25 mo
Time to diabetes onset (wk)	7	4	12	3	20	8	3	12	5	14
Diabetes antibodies	GADA (+) ICA (−)	GADA (+)	GADA (+) ICA (−) ZnT8 (−)	GADA (+) IA-2A (+)	GADA (−) ICA (−)	GADA (+) ICA (−) IA-2A (−)	GADA (+) IA-2A (+)	GADA (−) IA-2A (−)	GADA (+) ICA (−)	GADA (−) IA-2A (−) ZnT8 (−) IAA (−)
DKA	Yes	Yes	Yes	Yes	Yes	Yes	Yes	Yes	Yes	No
HbA1c (%)	7.5	9.2	7.8	8.4	8.3	NR	6.9	NR	8.2	6.9
C-peptide	NR	<10 ng/mL	86 pmol/L	0.02ng/mL	<0.01 µg/L	0.02 nmol/L	0.22 nmol/L	NR	Undetectable	0.24 ng/mL
HLA	–	–	–	–	–	DRB1*04-DQA1*03:01-DQB1*03:02	DRB1*04:16-DQB1*02:05-DQA1*01:03	DQB1*02/ DQB1 *06:02- DQA1 *01:02	–	DRB1*09:01-DQB1*03:03
Time to thyrotoxicosis onset (wk)	14	–	–	–	3 wk subclinical	8 wk subclinical	–	4	–	2
Time to hypothyroidism onset (wk)	23	4	16	9	12	next weeks	10 wk	UK	32	6
TD antibodies	TPOAb(−)	TPOAb(+)	TPOAb(−) TgAb(−)	TPOAb(+) TgAb(+) TRAb(+)	TPOAb(−)	TPOAb(−)	–	TPOAb(+) TgAb(−)	TPOAb(−)	TRAB(−) TPOAb(+) TgAb(+)
Therapeutic drug	L-T4	L-T4	L-T4 (50 µg/d)	L-T4 (200 µg/d)	L-T4 (125 µg/d)	L-T4	L-T4 (75 µg/d)	L-T4	L-T4 (50 µg/d)	L-T4 (100µg/d)
Other glands involved	–	–	–	–	–	–	–	Vitiligo	–	–
Other drugs	–	–	–	–	–	–	–	–	–	–

No.	21^[24]^	22^[24]^	23^[25]^	24^[25]^	25^[25]^	26^[26]^	27^[27]^	28^[28]^	29^[29]^	30^[30]^
Publication year	2018	2018	2018	2018	2018	2017	2016	2016	2016	2015
Gender	M	M	M	M	M	M	M	M	M	F
Age (yr)	83	65	82	23	61	63	65	54	58	66
Cancer type	Melanoma	Melanoma	SCC (oropharynx)	Melanoma	Melanoma	SCC (lung)	Lung adenocarcinoma	Melanoma	SCC (lung)	SCC (jaw)
ICI type	Pembrolizumab	Pembrolizumab	Pembrolizumab	Pembrolizumab	Ipilimumab+Pembrolizumab	Nivolumab	Ipilimumab + Pembrolizumab	Ipilimumab + Nivolumab	Pembrolizumab	PD-1
Previous medical history	Hypertension, right nephrectomy	–	–	–	–	–	T2DM	–	Hypertension	–
Previous medication history	–	–	Combined carboplatin/5-FU	–	–	Cisplatin	Metformin，glimepiride	–	Paclitaxel，carboplatingemcitabine	–
Time to diabetes onset (wk)	10	34	9	8	6	4	3	18	21	7
Diabetes antibodies	GADA (−) IA-2A (−) ZnT8(−)	GADA (−) IA-2A (−) ZnT8(−)	GADA (+) IA-2A (−) IAA (−) ZnT8 (−)	GADA (+) IA-2A (−) IAA (+)	GADA (+) IA-2A (−) IAA (−) ZnT8 (−)	GADA (+)	GADA (+) ICA (−) IAA (−) ZnT8 (−)	GADA (+)	IAA (−) ICA (−) GADA (−)	GADA (+) IA-2A (−) IAA (−) ZnT8 (−)
DKA	No	Yes	No	Yes	Yes	Yes	Yes	Yes	Yes	Yes
HbA1c(%)	9.4	8.5	8.5	7.5	8.6	7.2	8.5	NR	7.9	9.4
C-peptide	1 nmol/L	<0.1 nmol/L	<0.3 nmol/L	0.11 nmol/L	0.12 nmol/L	NR	undetectable	<0.1 ng/mL	0.03 nmol/L	<0.1ng/mL
HLA	DRB1*01:01-DQA1*01-DQB1*05:01/ DRB1*16:01-DQA1*01-DQB1*05:02	DRB1*04:01 DQA1*02 DQB1*02:02/ DRB1*07:01 DQA1*03 DQB1*03:01	DRB1*04-DQB1*03:02	DRB1*03/DRB1*04 DQB1*03:02	DRB1*03/ DRB1*15-DQB1*06	–	–	HLA-I A2 and HLA-II DQB1*06:02	DRB1*09:01-DQB1*03:03/ DRB1*14:05-DQB1*05:03	DR3-DQ2/DR4-DQ8
Time to thyrotoxicosis onset (wk)	HT	HT	–	8 wk thyroiditis	24 wk thyroiditis	–	3	2	21 wk subclinical	–
Time to hypothyroidism onset (wk)			UK	UK		12	35	9	–	7
TD antibodies	TPOAb(+)	TPOAb(+)	–	–	–	TPOAb(+)	TPOAb(+)	TRAb(+)	TRAb(−) TPOAb(−)	TPOAb(+)
Therapeutic drug	–	–	–	–	–	L-T4	L-T4 (1.7 mg/kg/d)	L-T4	–	–
Other glands involved	Maculopapular rash	–	Rash, arthriti	Rash, inflammatory polymyopathy	Rash	–	–	Rash, tachycardia, and reports of hot flashes，ICI-induced hypophysitis	–	–
Other drugs	–	–	–	–	–	–	–	prednisone and metoprolol	–	–

Abbreviations: – = not report, HC = hydrocortisone, HT = Hashimoto thyroiditis, IA-2A = islet antigen-2 antibody, UK = it happened but the specific time is unknown, ZnT8 = Zinc transporter 8.

## 4. Discussion

### 4.1. Summary of the results

The median age of the collected patients was 61 years (range 23–85 years), with a male predominance of 63.3%. Melanoma and lung cancer constituted the most frequent malignancies, comprising 40% and 30% of confirmed cases, respectively, both historically prioritized for ICI therapies due to earlier regulatory approvals. Other tumor types included esophageal cancer, rectal cancer, oropharyngeal squamous cell carcinoma, and others.

Over half of the patients received programmed cell death protein 1 (PD-1) inhibitors, including nivolumab, toripalimab, camrelizumab, and pembrolizumab. Three patients received programmed death-ligand 1 (PD-L1) inhibitors. Five patients received PD-1 combined with cytotoxic T-lymphocyte antigen 4 (CTLA-4) inhibitors: 3 with ipilimumab-nivolumab and 2 with ipilimumab-pembrolizumab.

Due to inconsistent descriptions of the timing of adverse reactions in each case report, we organized the cases on a weekly basis. A summary of the results can be found in Table 5. The 30 cases of ICI-DM occurred between 3 to 45 weeks after treatment initiation (median 8 weeks), with 23 out of 30 patients presenting with DKA at diabetes onset. The following requires clinical attention: although ICI-induced DKA mostly presents with gastrointestinal symptoms, some patients may exhibit only polyuria, thirst, fatigue, agitation, or blurred vision but still develop severe DKA.^[1,16,22,26]^ Additionally, ICI-DM can sometimes be difficult to control, with patients experiencing recurrent DKA even under insulin management.^[1,26]^

**Table 5:** Characteristics of ICI-induced thyroid dysfunction and diabetes (n = 30).

Characteristic	All cases (n = 30)
Age, yr	
Median (range)	61 (23–85)
Gender	
Female/male	11 vs 19
Tumor types	
Melanoma	12/30 (40%)
Lung cancer	9/30 (30%)
Other cancers	9/30 (30%)
Immune checkpoint inhibitor	
PD-1 inhibitor	22/30 (73.3%)
PD-L1 inhibitor	3/30 (10%)
PD-1 + CTLA-4 inhibitors	5/30 (16.7%)
Time to diabetes onset, wk	
Median (range)	8 (3–45)
GADA pos./GADA neg. (median)	6 vs 14
Diabetic ketoacidosis	23/30 (76.7%)
GADA pos./GADA neg.	88.2% vs 61.5%
Hemoglobin A1c, median (range)	7.9% (5.6%–9.4%)
Low or undetectable C-peptide	26/30 (86.7%)
Elevated lipase	1/30 (3.3%)
Elevated amylase	1/30 (3.3%)
Diabetes antibodies	
GADA (+)	18/30 (60%)
IAA (+)	2/30 (6.7%)
IA-2A (+)	2/30 (6.7%)
ICA (+)	0
ZnT8 (+)	0
Hypothyroidism following thyrotoxicosis	11/30 (36.7%)
PD-1 inhibitor	6/11 (54.5%)
PD-L1 inhibitor	1/11 (9.1%)
PD-1 + CTLA-4 inhibitors	4/11 (36.4%)
Hypothyroidism alone	11/30 (36.7%)
Thyrotoxicosis alone	5/30 (16.6%)
Not Report	3/30 (10%)
TD antibodies	
TPOAb(+)	16/30 (53.3%)
TgAb(+)	6/30 (20 %)
TRAb(+)	3/30 (10 %)
The sequence of occurrence	
ICI-DM before ICI-TD	7/30 (23.3%)
ICI-TD before ICI-DM	7/30 (23.3%)
ICI-DM and ICI-TD concurrent	13/30 (43.4%)
Not Report	3/30 (10 %)

Abbreviations: CTLA-4 = cytotoxic T-lymphocyte antigen 4, GADA = glutamic acid decarboxylase antibody, IA-2A = islet antigen-2 antibody, IAA = insulin autoantibody, ICA = islet cell antibody, ICI-DM = ICI-induced diabetes mellitus, ICI-TD = ICI-induced thyroid dysfunction, PD-1 = programmed cell death protein 1, PD-L1 = programmed death-ligand 1, TgAb = thyroglobulin antibody, TPOAb = thyroid peroxidase antibody, TRAb = thyroid stimulating hormone receptor antibody, ZnT8 = Zinc transporter 8.

Based on the rapid onset of the disease, ICI-DM is primarily characterized by FT1DM. At diagnosis, the median HbA1c level was 7.9% (range 5.6%–9.4%), indicating only mild elevation, even within the normal range, which contrasts with the hyperglycemic symptoms observed. Nearly all ICI-DM patients exhibited reduced or undetectable C-peptide levels, distinguishing it from classic type 1 diabetes (T1DM), where C-peptide is detectable in approximately 93% of patients within the first 2 years of onset. Regarding autoantibodies, 40% of patients were autoantibody-negative, while 60% tested positive for glutamic acid decarboxylase antibodies (GADA), the predominant autoantibody, although the detection rate of this antibody was still lower than the 80% reported in classic T1DM.

Although organ-specific antibodies like GADA may be involved in irAEs, their exact role remains unclear. Rather than being a direct cause of diabetes, GADA may instead reflect an underlying autoimmune predisposition.^[31]^ Interestingly, previous studies^[19]^ support our findings, by showing that GADA positivity is associated with an earlier onset of ICI-DM (median 6 weeks vs 14 weeks) and a higher risk of DKA (88.2% vs 61.5%), suggesting that GADA may serve as a marker of more aggressive or advanced immune dysregulation.

Eleven patients developed hypothyroidism subsequent to thyrotoxicosis, 4 of which were associated with PD-1 and CTLA-4 inhibitors. Of course, we only collected 5 patients who were receiving dual ICI therapy. This supports the increased risk of transitioning from thyrotoxicosis to hypothyroidism with combination therapy.^[32]^ Additionally, 11 cases presented with hypothyroidism alone, while only 5 cases showed thyrotoxicosis (2 without long-term follow-up). Previous study has suggested that ICI-induced overt thyrotoxicosis (with or without hypothyroidism) and hypothyroidism (without thyrotoxicosis) may have distinct etiologies, rather than being different manifestations of the same disease process.^[32]^ Two patients^[6,9]^ who only developed hypothyroidism clearly tested positive for antibodies before treatment, which somewhat supports the view that hypothyroidism (without thyrotoxicosis) may be accelerated by ICI treatment for preexisting subclinical Hashimoto thyroiditis (HT), rather than ICI-induced new thyroiditis. Interestingly, the patient in our case was also TPOAb positive before treatment, but after treatment, we observed a change from thyrotoxicosis to hypothyroidism. In our summary of cases, we did find that the incidence of extrathyroid irAEs, such as primary adrenal insufficiency, secondary adrenal insufficiency, hypophysitis, and vitiligo, was significantly higher in patients with overt thyrotoxicosis (with or without transient hypothyroidism) compared to those with hypothyroidism. Thyroid dysfunction was detected in 7 cases before the onset of diabetes, 7 cases afterward, and concurrently with diabetes in 13 cases, with other 3 cases not reported. Thyrotoxicosis and hypothyroidism induced by ICIs often present asymptomatically or with fatigue similar to that caused by antitumor treatments, making them easily overlooked and detected concurrently with diabetes. This can result in cases where thyrotoxicosis is initially missed, potentially leading to an increase in cases presenting solely as hypothyroidism over time.

Early elevation of serum thyroglobulin (Tg)^[33]^ and preexisting Tg autoimmunity have been linked to PD-1 inhibitors–induced destructive thyroiditis.^[34]^ Baseline thyroglobulin antibody (TgAb) or TPOAb positivity and irregular thyroid ultrasound findings are considered potential risk factors for ICI-TD,^[32,35–37]^ though the role of these antibodies remains debated. Some studies^[35]^ identify TgAb as an independent risk factor, while others^[38]^ suggest TPOAb may predict hypothyroidism following thyrotoxicosis. However, some studies have indicated that there is no association between baseline TPOAb and TgAb levels and the risk of ICI-TD.^[39]^ Currently, it remains inconclusive whether elevated thyroid autoantibodies are the causative factor of thyroid dysfunction or a humoral immune response triggered by the release of thyroid antigen during the development of destructive thyroiditis.^[40]^ However, conflicting evidence exist,^[41,42]^ and many ICI-TD patients lack detectable thyroid autoantibodies, implying alternative or antibody-independent mechanisms. Seventeen cases were positive for thyroid autoantibodies. However, due to the lack of detailed information on the actual progression of thyroid diseases, it is difficult to accurately determine the early onset of thyroid diseases in thyroid autoantibodies positive patients. Further prospective studies are required to draw conclusive results.

### 4.2. The pathogenesis of ICI-DM and ICI-TD

The etiology of ICI-DM and ICI-TD remains incompletely understood, though T cell-mediated immunity is likely central. PD-1 possesses a protective effect within the human body,^[43]^ and its expression in CD4+ T cells and Tregs is notably reduced in diabetic individuals. Similarly, diminished CTLA-4 expression has been observed in FT1DM,^[44]^ suggesting that both checkpoints critically regulate disease progression. Preclinical studies reveal PD-1 inhibition accelerates diabetes onset across age groups, while CTLA-4 inhibition primarily affects young non-obese diabetic mice,^[45]^ indicating distinct roles: CTLA-4 regulates naïve T cells, whereas PD-1 controls both naïve and autoimmune T cell activation. In a mouse model of diabetes, depleting CD8+ T cells temporarily cured the disease, while depleting CD4+ T cells failed to halt its progression.^[34]^ This suggests that activated autoimmune T cells are predominantly CD8+ T cells, consistent with the observation of specific IFN-γ CD8+ T cells infiltrating the islets and almost completely destroying islet β cells in hyperglycemic mice treated with PD-L1 antibodies. β cells themselves protect against detrimental crosstalk with proinsulin-specific CD8+ T cells. Changes in the β cell environment, such as a selective deficiency of PD-L1 on target cells or the absence of PD-1 on T cells, may render pancreatic β cells more vulnerable to attacks by proinsulin-specific CD8+ T cells.^[46]^ Additionally, PD-1 inhibitors can inhibit T cell migration, prolonging interaction time, promoting the coupling of T cells and major histocompatibility complex (MHC), enhancing T cell activation, and triggering cell-mediated autoimmune diabetes.^[47]^ This can result in a sudden decrease in insulin storage, leading to DKA.

Cell-mediated cytotoxicity appears to have no significant impact on α cells and exocrine glands.^[17]^ Intriguingly, although glucagon levels remain stable, α cell responsiveness appears reduced.^[24]^ Elevated lipase and amylase in 2 ICI-DM patients^[7,19]^ suggest possible exocrine involvement, and some studies have reported antibodies against exocrine enzymes.^[48]^ However, nonspecific enzyme elevation without diabetes has also been observed. Hence, further assessment of exocrine gland and α cell damage is requisite.

Consistent with ICI-DM findings, increased CD8+ T cell infiltration in affected organs plays a crucial role in the development of ICI-TD. Kobayashi^[37]^ identified that destructive thyroiditis is the main cause of PD-1 inhibitors induced thyroid dysfunction. Elevated levels of IL-2 (a Th1 cytokine) and reduced levels of MCP-1 (a Th2 chemokine) in the ICI-TD group indicate an increase in the Th1/Th2 balance,^[33]^ consistent with the Th1 imbalance observed in HT.^[49]^ CD8+ T cells activated by IL-2 may destroy immune tolerance, targeting not only tumor cells but also attacking thyroid cells, leading to ICI-TD.

Interestingly, CD4+ T cells also play a crucial role in PD-1 inhibitors induced destructive thyroiditis. In a thyroglobulin-preimmunized mouse model of thyroid irAEs, Yasuda et al^[34]^ observed thyroidal infiltration of CD4+ T cells. Depletion of CD4+ T cells completely prevented disease progression, whereas CD8+ T cell depletion only partially did so, suggesting that Tg-specific cytotoxic CD4+ T cells may directly damage thyroid follicular cells via MHC Class II and regulate CD8+ T cell cytotoxicity.

There is no consensus on whether B-cell-mediated humoral immunity is involved in the development of ICI-DM and ICI-TD. Yasuda et al^[34]^ propose that humoral immunity is unlikely to play a predominant role in PD-1 inhibitors induced destructive thyroiditis, as depletion of CD20 B cells in mice did not prevent the progression. Moreover, DKA can occur without GADA, indicating that cell-mediated immune activation alone can occur without humoral immunity shortly before clinical presentation.^[23]^ However, opposing evidence exists: B cells and tertiary lymphoid structures have been observed in the tumors of ICI responders.^[33]^ What’s more, although the number of Graves’ disease patients induced by ICI is relatively rare, our summary table includes 1 case that may have been caused by the activation of B cells and plasma cells by CD4+ T helper cells, leading to the production of thyroid stimulating hormone receptor antibodies.^[13]^

### 4.3. Human leukocyte antigen associated with autoimmune diseases

Currently, it is widely believed that HLA is a genetic background element associated with autoimmune diseases, playing a crucial role in immune responses. The genetic polymorphism of HLA is closely linked to variations in the intensity of immune responses to foreign antigens.^[5]^ Immunotherapy may accelerate disease progression in individuals carrying diabetes-predisposing HLA alleles.^[50]^

The presence of HLA-DR3 and/or DR4, especially with HLA-DQ3, indicates shared susceptibility to T1DM and thyroid autoimmunity.^[5]^ HLA haploid analysis was conducted on 15 patients with ICI-DM and ICI-TD, and susceptibility genes were identified in twelve patients. Specific haplotypes – DRB1*08:02-DQA1*04:01–DQB1*04:02 and DRB1*09:01–DQA1*03:02–DQB1*03:03 – are more frequent in HT and T1DM patients than in controls, and were identified in 3 patients.^[51]^ Interestingly, although DQB1*06 is considered protective in some autoimmune diseases.^[21,28]^ It was found in 3 patients, including 1 with both DR3 and DQ6, suggesting that protective haplotypes may not be superior to susceptible haplotypes in ICI-related irAEs.

A Japanese study^[52]^ found DRB1*09:01–DQB1*03:03 predominant in GADA-positive FT1DM, and DRB1*04:05–DQB1*04:01 in GADA-negative FT1DM. However, Among the 3 patients with the aforementioned haplotypes that we know of, 2 do not support this view. Therefore, HLA typing alone is not a reliable method for predicting ICI-related irAEs in clinical practice.

### 4.4. ICI-TD and ICI-DM caused by sintilimab

Sintilimab is a fully humanized IgG4 monoclonal antibody targeting PD-1, exhibiting a mechanism of action similar to nivolumab and pembrolizumab while demonstrating higher affinity for human PD-1.^[53]^ Initially approved by China in 2018, sintilimab was later evaluated in the phase III ORIENT-16 trial involving patients with unresectable locally advanced or metastatic gastric and gastroesophageal junction adenocarcinoma. This study demonstrated that sintilimab plus chemotherapy significantly improved overall survival for all patients and for patients with a combined positive score of 5 or more compared with placebo plus chemotherapy.^[54]^ At present, the combination of sintilimab with chemotherapy has been established as a first-line treatment option for gastric cancer across all patient subgroups.

Following sintilimab treatment, the patient sequentially developed ICI-TD and ICI-DM, both classified as irAEs in the ORIENT-16 study. According to the latest prescribing information summarizing clinical trial data, the incidence of hypothyroidism and hyperthyroidism was 17.4% and 8.9%, respectively, while the incidence of hyperglycemia and T1DM was 1.3%, with a median time to onset of 105 days (range: 9–494 days). The prescribing information also notes that irAEs may occur during treatment or after discontinuation.

This case demonstrates several distinctive features. To begin with, this represents a rare case of sintilimab-induced ICI-DM and ICI-TD. In addition, ICI-DM does not occur during sintilimab. The delayed-onset of adverse events after treatment cessation is consistent with the findings of Couey et al,^[4]^ who reported that over half of DIRE cases occured following short courses of immunotherapy with ≤4 treatment cycles, which matches the treatment course of our patient. Furthermore, although Seo et al^[55]^ have reported delayed-onset diabetes occurring 4 to 6 months after therapy with nivolumab, atezolizumab, and ipilimumab, this is the first documented case associated with sintilimab, with onset more than 4 months after discontinuation. The mechanism may involve sustained PD-1 receptor occupancy on T cells following treatment discontinuation, as previous studies have demonstrated up to 40% receptor occupancy persisting 8 months after cessation of nivolumab therapy.^[56]^

Although concurrent medications (tegafur, oxaliplatin, and apatinib) were administered, literature review excluded their causal roles. Tegafur and oxaliplatin lack reported endocrine toxicity, while apatinib’s prescribing information describes hyperglycemia but not FT1DM. The hyperglycemia induced by it may be associated with insulin resistance.

As for thyroid dysfunction, the most recent hospital evaluation of the patient revealed that the hypothyroidism had persisted for over 5 months. It has been 3 months since apatinib was discontinued, which aligns more closely with the timeline of ICI-TD. Furthermore, thyroid dysfunction induced by ICIs are often challenging to reverse. The patient experienced a transition from thyrotoxicosis to hypothyroidism, with the pathological process more closely resembling the primary mechanism of ICI-TD, which is destructive thyroiditis. We found reports of sunitinib being associated with lymphocytic thyroiditis,^[57]^ but current studies with the largest number of participants on apatinib-induced hypothyroidism have not clearly documented this process.^[58]^ The temporal association and pathological evidence strongly implicate that sintilimab is the causative factor.

Routine monitoring during ICIs administration enables early detection of most adverse events. However, for patients with a history of ICI exposure, clinicians must remain vigilant about the potential emergence of DIRE, even months after discontinuing ICI therapy. In the event of disease occurrence, it is essential to consider not only recent treatments and interventions but also to carefully differentiate DIRE from other conditions to avoid misattribution. Misattribution may lead to delayed treatment or inappropriate discontinuation of beneficial therapies. It is also recommended that drug prescribing information for ICIs explicitly include a specified period of continued monitoring following drug discontinuation.

## 5. Limitations

This retrospective case study has inherent limitations due to incomplete data entries, which could potentially confound the causality assessment between ICIs and irAEs. Furthermore, the absence of postdischarge follow-up data precludes longitudinal observation of disease progression and prognostic evaluation. Existing literature suggests that previous treatment with TKIs appears to be a significant risk factor for developing hypothyroidism during PD-1 inhibitors therapy,^[59]^ whereas no such association was found with previous chemotherapy.^[60]^ Notably, our current hypothesis cautiously attributes the observed thyroid dysfunction primarily to sintilimab in this case, though future large-scale prospective studies are warranted to validate this causal relationship.

## 6. Conclusion

We present a rare case of sintilimab-induced thyroid dysfunction and diabetes mellitus. Our case suggests that ICI-DM can occur not only during the period of medication but also within a certain period after discontinuation of the sintilimab.

We reviewed previous case reports to elaborate on the clinical manifestations, autoantibodies, potential pathogenesis, and reported factors influencing the prediction of these 2 endocrine irAEs, with the aim of enhancing clinicians’ awareness.

## Author contributions

**Data curation:** Wenxue Zhang, Xiaolu Gao.

**Investigation:** Ying Zan, Wenxue Zhang.

**Methodology:** Yedong Wei.

**Writing – original draft:** Ying Zan, Yedong Wei, Xiaolu Gao.

**Writing – review & editing:** Ying Zan, Jigang Si.
